# Sub-Acute Toxicity Study of Tiger Milk Mushroom *Lignosus tigris* Chon S. Tan Cultivar E Sclerotium in Sprague Dawley Rats

**DOI:** 10.3389/fphar.2016.00246

**Published:** 2016-08-09

**Authors:** Boon-Hong Kong, Nget-Hong Tan, Shin-Yee Fung, Jayalakshmi Pailoor

**Affiliations:** ^1^Medicinal Mushroom Research Group, Department of Molecular Medicine, Faculty of Medicine, University of MalayaKuala Lumpur, Malaysia; ^2^Department of Pathology, Faculty of Medicine, University of MalayaKuala Lumpur, Malaysia

**Keywords:** *Lignosus tigris*, tiger milk mushroom, sclerotium, sub-acute toxicity, Sprague-Dawley rats

## Abstract

*Lignosus* also known as “Tiger Milk Mushroom,” is classified in the family Polyporaceae and mainly consumed for its medicinal properties in Southeast Asia and China. The sclerotium is known as the part with medicinal value and often used by the natives to treat a variety of ailments. *Lignosus tigris* Chon S. Tan, one of the species of the Malaysia Tiger Milk mushroom, has recently been successfully cultivated in laboratory. Earlier studies have demonstrated the *L. tigris* cultivar E sclerotia exhibited beneficial biomedicinal properties. This study evaluated the potential toxicity of *L. tigris* E sclerotia in a 28-day sub-acute oral administration in Sprague Dawley (SD) rats. *L. tigris* E sclerotial powder was administered orally at three different doses of 250, 500, and 1000 mg/kg to the SD rats once daily, consecutively for 28-days. Body weight of the rats was recorded and general behavior, adverse effects, and mortality were observed daily throughout the experimental period. At the end of the experiment, blood hematology and biochemistry, relative organ weights, and histopathological analysis were performed. Results showed that there were no mortality nor signs of toxicity throughout the 28-day sub-acute toxicity study. Oral administration of the *L. tigris* E sclerotial powder at daily dose up to 1000 mg/kg had no significant effects in body weight, relative organ weight, blood hematological and biochemistry, gross pathology, and histopathology of the organs. *L. tigris* E sclerotial powder did not cause any treatment-related adverse effect in the rats at different treatment dosages up to 1000 mg/kg. As the lethal dose for the rats is above 1000 mg/kg, the no-observed-adverse-effect level (NOAEL) dose is more than 1000 mg/kg.

## Introduction

*Lignosus* spp. belonging to the Polyporaceae family has been long valued for its medicinal and functional properties. The species of the *Lignosus* mushroom including *L. dimiticus* Ryvarden, *L. ekombitii* Douanla-Meli, *L. goetzii* (Henn.) Ryvarden, *L. hainanensis* B. K. Cui, *L. sacer* (Afzel. ex Fr.) Ryvarden and *L. rhinocerotis* (Cooke) Ryvarden. *L. rhinocerotis* (commonly known as the Tiger Milk Mushroom) is utilized by the local communities in Southeast Asia and China as traditional medicine. The sclerotium is the part with medicinal values which is used as general tonic and natural remedy for various ailments.

In Peninsular Malaysia, the mushroom sclerotia was utilized by Orang Asli (Semai, Temuan, and Jakun) for treating variety of illnesses including coughs, asthma, food poisoning, breast swelling, arthritis, edema, liver diseases, and cancer (Lee et al., 2009; Ismail, 2010). Local Malay and Chinese traditional practitioners have used it to treat leukemia, cervical cancer, intestinal ulcer disease, kidney disease, body swelling, fever, asthma, and consumed as general tonic (Tan, 2009). In Hong Kong and China, the mushroom sclerotial is a costly traditional medicine utilized for the treatment of gastric ulcers and liver-related diseases, such as hepatitis and cancer (Wong and Cheung, 2009). Recent scientific studies have proved that *L. rhinocerotis* has a wide spectrum of bioactive properties. An *in vitro* study on the antioxidant activity revealed that the mushroom sclerotial extracts exhibited potent antioxidant activity (Yap et al., 2013). Lee et al. (2012) demonstrated that the cold water extract of *L. rhinocerotis* sclerotium exhibited significant anti-proliferative activity against human breast (MCF-7), and lung (A549) adenocarcinoma cells. The polysaccharides of the *L. rhinocerotis* sclerotium were reported to have immunomodulatory effect by activating human innate immune cells (Wong et al., 2011). The sclerotial extracts of the *L. rhinocerotis* have also been reported to have acute anti-inflammatory activity and antimicrobial properties against bacteria (Mohanarji et al., 2012; Lee et al., 2014). It also has the ability to enhance neurite outgrowth in rat adrenal pheochromocytoma (PC-12) cells (Eik et al., 2012). Owing to its wide ethnomedicinal usages and supportive data on its medicinal properties, *L. rhinocerotis* is potentially to be developed as health supplement. Toxicity assessments including sub-acute, chronic, fertility, teratogenic, and mutagenic studies on *L. rhinocerotis* sclerotial powder all indicated it is safe for human consumption (Lee et al., 2011, 2013).

Recently, a new species of *Lignosus* mushroom collected from the tropical forest in the state of Pahang, Malaysia has been identified and named *Lignosus tigris* Chon S. Tan (Tan et al., 2013). The sclerotia of this mushroom have also been used as traditional medicine by the local community. It is not distinguished from *L. rhinocerotis* due to its similarity in gross morphologies (Tan et al., 2013). Yap et al. (2014a) revealed that the wild type *L. tigris* possess good nutritive value and exhibited potent antioxidant activity. Recently, *L. tigris* mushroom has been successfully cultivated by Ligno Biotech (Malaysia).

In our earlier study, the sclerotia of the *L. tigris* cultivar E has been shown to have high carbohydrate, protein and dietary fiber content (Kong et al., 2016). The sclerotia also contain high amount of potassium which is an essential electrolytes to maintain body function. Substantial amount of essential amino acids was found in the sclerotial protein. It also possesses potent superoxide anion scavenging activity. The mushroom's sclerotial extracts show high contents of glucan and terpenoid, compounds which may exhibit anticancer and immunomodulatory properties (Huang et al., 2012; Kong et al., 2016). The study suggested that *L. tigris* E sclerotia is a promising medicinal mushroom and may also be a good source of functional food and nutraceutical. Therefore, the toxicity data of the mushroom is essential before it being developed as health supplement. Acute toxicity assessment has indicated that *L. tigris* E sclerotial powder did not exhibit acute toxicity at the dose of 2000 mg/kg. In this study, we examined the sub-acute toxicity of the *L. tigris* cultivar E to investigate the potential toxicity of repeated exposure of the mushroom sclerotial powder in Sprague-Dawley rats.

## Materials and methods

### Cultivation and preparation of *L. tigris* E sclerotial powder

Wild type of *L. tigris* Chon S. Tan specimen was collected from a tropical forest in Lata Iskandar (4°17.46′N 101°34.41′E), Pahang, Malaysia (Tan et al., 2013). The fungus identity was verified by PCR amplification coupled with sequence analysis of the internal transcribed spacer (ITS) region (Yap et al., 2014b). Live culture of the fungus was prepared inoculating a small portion (50 mg) of clean sclerotial tissue in potato dextrose agar (PDA) supplemented with 100 μg/L of streptomycin. Pure and healthy mycelium culture (200 mg) was then inoculated in sterilized 100 g of brown rice with 100 ml of distilled water in a polypropylene container. The inoculated brown rice was incubated in dark at 24°C for 3 months. The sclerotia grown from the surface of rice media were then harvested and freeze dried. The freeze dried sclerotia were ground into powder using 0.2 μm mesh filter. The nutritional and amino acid compositions of the sclerotial powder have been analyzed in our previous study. The sclerotium is rich in carbohydrate, protein, dietary fibers, minerals and contains considerable amount of essential amino acids and bioactive components, such as phenolics, terpenoids, and glucan (Kong et al., 2016).

### Experimental animals

A total of 80 (40 males and 40 females) Sprague-Dawley (SD) rats aged 7–8 weeks old were obtained from the Animal Experimental Unit, University of Malaya. The experimental rats were housed under the conditions with a 12 h of light/dark cycle and temperature adjusted between 22 and 25°C with 60 ± 10% of humidity. Rats were given free access to standard sterile rodent diet (Altromin #1324 Rodent Maintenance Diet, Denmark) and water. The experimental procedures were approved by the Institutional Animal Care and Use Committee, University of Malaya (UM IACUC—Ethics reference no. 2013-09-17/MOL/R/TNH).

### Dose formulation and administration

The sub-acute toxicity study was performed according to the guidelines of Organization for Economic Co-operation and Development (OECD) for testing of chemicals, TG 407 (adopted—3 October 2008; OECD, 2008). SD rats were assigned into four groups; a control group and 3 treatment groups (*n* = 10; 5 males and 5 females). *L. tigris* E sclerotial powder was dissolved in distilled water and administered orally on a daily basis for 28-days at single doses of 250, 500, and 1000 mg/kg with the volume not exceeding 1 ml/100 g. Rats in control group received only distilled water (1 ml/100 g).

### Observations, examinations, and measurements

The behavioral changes of the rats were observed at least once daily for mortality and signs of toxicity. The body weight of each rat was measured on the first day of treatment and weekly thereafter.

### Blood hematological and biochemical analyses

Rats were fasted overnight at the end of 28-days and anesthetized with ketamine (55 mg/kg) and xylazine (5 mg/kg). Blood samples were withdrawn through cardiac puncture. Hematological and biochemical tests were performed based on the parameters including red blood cell (RBC) count, hemoglobin concentration, packed cell volume (PCV), mean corpuscular volume (MCV), mean corpuscular hemoglobin (MCH), mean corpuscular hemoglobin concentration (MCHC) along with platelet, white blood cell (WBC), neutrophil, lymphocyte, monocyte, eosinophil, basophil, atypical lymphocyte counts, glucose, urea, creatinine, calcium, inorganic phosphate, uric acid, sodium, potassium, chloride, total cholesterol, total protein, albumin, globulin, albumin globulin ratio, total bilirubin, alkaline phosphatase, serum glutamic oxaloacetic transaminase (SGOT or AST), and serum glutamic pyruvic transaminase (SGPT or ALT) (Lee et al., 2013).

### Histopathological study

After blood collection, rats were euthanized by anesthetic overdose of 200 mg/kg sodium pentobarbital. Selected vital organs (heart, lungs, liver, spleen, and kidneys) and reproductive organs (uterus, ovaries, prostate, testes, and seminal vesicles) were excised, weighed, and fixed in 10% buffered formalin with the exception of the testes which were fixed in modified Davidson's fluid. The relative organ weight was calculated as (organ/body weight) × 100%. After fixation, the tissues were subjected to hematoxylin and eosin staining. Signs of toxicity in the selected organs were observed by light microscopic examinations. Changes in the cardiac muscle fibers, such as degeneration, inflammation, and necrosis, and lung tissue including inflammatory exudate in the alveolar or interstitial spaces and degeneration were inspected. Other toxicity parameters, such as necrosis of renal tubules or glomerular; changes in liver architecture, fat content and congestion in hepatocytes, evidence of cholangitis and occurrence of inflammatory cell infiltrate in the portal tracts; and alterations of spleen architecture or signs of infection were observed (Kong et al., 2016). Histologic structures of the female and male reproductive organs of the rats were also examined.

### Statistical analysis

Statistical analysis was performed using IBM SPSS (Statistical Package for Social Sciences) Statistics 22. All data were expressed as means ± standard error of the mean (SEM). Dunnett *t* (two-sided) test was used to determine the statistical differences between the means of control and treatment groups. Dunnett T3 test was used on condition of variance heterogeneity. Levene statistics was used to calculate the homogeneity of variances. All differences were considered statistically significant at the *p* < 0.05 level.

## Results

### General findings

**Figure 1:**
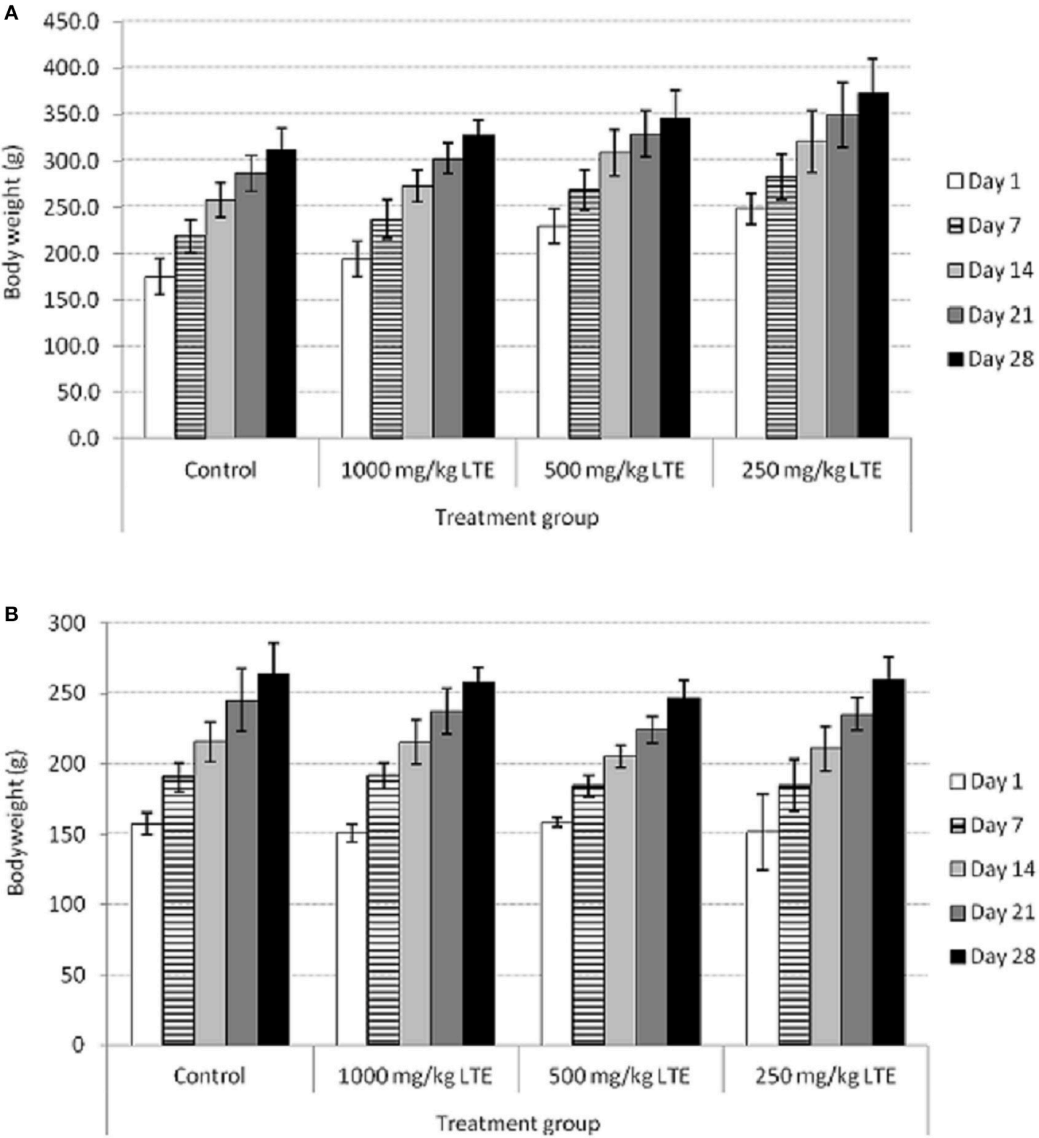
**Body weight of control and treated (A) male and (B) female rats with 250, 500, and 1000 mg/kg of *L. tigris* E (LTE) sclerotial powder for 28-days**. Body weight is shown as mean ± SD (*n* = 5).

The oral administration of *L. tigris* E sclerotial powder at different doses did not produced any mortality or abnormal clinical signs. There were no abnormal changes in body weights in either sex caused by the oral administration of the *L. tigris* E sclerotial powder (Figure 1). Normal body weight gain was observed in both male and female rats of all the treated and control groups throughout the experimental period (Table 1).

**Table 1:** **Body weight gain of male and female rats treated with *L. tigris* E sclerotial powder for 28-days**.

**Treatment**	**Body weight gain (g)**							
	**Male**				**Female**			
	**Day 7**	**Day 14**	**Day 21**	**Day 28**	**Day 7**	**Day 14**	**Day 21**	**Day 28**
Control	43.5 ± 4.7	82.4 ± 3.6	111.7 ± 7.5	136.1 ± 8.0	33.3 ± 7.7	58.6 ± 13.7	87.9 ± 20.9	106.9 ± 20.6
1000 mg/kg	43.0 ± 2.0	78.6 ± 4.6	108.7 ± 11.3	134.0 ± 13.4	41.4 ± 3.2	64.9 ± 10.2	87.1 ± 13.4	107.2 ± 9.3
500 mg/kg	39.1 ± 5.0	79.7 ± 8.1	99.6 ± 9.5	117.1 ± 14.5	25.6 ± 5.2	46.9 ± 6.0	65.8 ± 6.8	88.1 ± 11.3
250 mg/kg	34.2 ± 9.6	72.7 ± 18.4	101.6 ± 22.0	124.6 ± 24.6	32.7 ± 13.1	58.9 ± 18.0	83.5 ± 22.4	108.4 ± 23.4

Values are expressed in mean ± SD (n = 5/group). No significance difference in the body weight gain was observed between the control and treated rats (p > 0.05).

### Blood hematological and biochemical parameters

The effects of daily administration of *L. tigris* E sclerotial powder for 28 consecutive days on hematological and biochemical parameters are presented in Tables 2, 3. A few statistically significant differences between control and treated groups of the male and female rats were noted with respect to both hematological (Table 2), and biochemical (Table 3) parameters. Decreasing MCV, MCH, and neutrophil counts were noted in low (250 mg/kg) and middle (500 mg/kg) doses treated male rats. MCV level in males of the both 250 and 500 mg/kg groups were significantly lower than the control group. MCH level and neutrophil counts in males of the 250 and 500 mg/kg groups, respectively, were also significantly lower than the control group. Significant differences for RBC, hemoglobin, PCV, MCH, MCHC, platelet, WBC, and lymphocyte counts were observed in the treated female rats. Both 250 and 500 mg/kg treated female groups exhibited significantly higher RBC and lower MCH and MCHC than the control group. Hemoglobin level in females of the 250 and 1000 mg/kg groups were significantly higher than the control group. The PCV level in all the treated female groups was also significantly higher compared to control group. The 250 mg/kg group of the female rats showed higher platelet and WBC counts than the control group (*p* < 0.05). Similarly, the female rats in 1000 mg/kg group showed higher lymphocyte counts (*p* < 0.05) than the control group.

**Table 2:** **Hematological values of male and female rats treated with 250, 500, and 1000 mg/kg of *L. tigris* E sclerotial powder for 28-days**.

**Parameter**	**Treatment**			
	**Control**	**1000 mg/kg**	**500 mg/kg**	**250 mg/kg**
**MALE**				
RBC (X 10^12^/L)	7.50 ± 0.46	7.56 ± 0.11	8.00 ± 0.22	8.54 ± 0.77
Hemoglobin (G/DL)	15.98 ± 0.75	15.64 ± 0.49	15.98 ± 0.30	16.2 ± 1.15
PCV (%)	48.00 ± 2.65	47.60 ± 0.55	49.40 ± 0.55	48.80 ± 2.68
MCV (FL)	64.20 ± 2.17	62.60 ± 0.89	61.40 ± 1.95^**^	57.20 ± 1.48^**^
MCH (PG)	21.20 ± 0.84	20.60 ± 0.89	20.00 ± 0.71	19.00 ± 0.00^**^
MCHC (G/DL)	33.20 ± 1.79	33.00 ± 1.41	32.40 ± 0.55	33.20 ± 0.45
Platelet count (X 10^9^/L)	827.40 ± 138.51	935.40 ± 163.05	981.80 ± 100.36	926.80 ± 93.71
WBC (X 10^9^/L)	9.38 ± 2.01	11.14 ± 2.78	10.48 ± 1.71	10.48 ± 1.47
Neutrophil (%)	47.40 ± 12.16	32.00 ± 15.52	19.40 ± 3.65^**^	26.20 ± 7.09
Lymphocyte (%)	44.60 ± 17.34	62.80 ± 18.7	76.20 ± 4.32	68.40 ± 6.73
Monocyte (%)	7.80 ± 5.72	5.00 ± 4.12	4.40 ± 1.52	5.20 ± 2.17
Eosinophil (%)	0.00 ± 0.00	0.00 ± 0.00	0.00 ± 0.00	0.00 ± 0.00
Basophil (%)	0.00 ± 0.00	0.00 ± 0.00	0.00 ± 0.00	0.00 ± 0.00
Atypical lymphocyte (%)	0.20 ± 0.45	0.20 ± 0.45	0.00 ± 0.00	0.20 ± 0.45
**FEMALE**				
RBC (X 10^12^/L)	6.44 ± 0.54	7.12 ± 0.28	7.80 ± 0.42^**^	8.40 ± 0.48^**^
Hemoglobin (G/DL)	13.44 ± 1.11	14.94 ± 0.45^**^	14.70 ± 0.69	16.30 ± 0.71^**^
PCV (%)	39.20 ± 2.39	43.60 ± 1.14^**^	46.00 ± 2.35^**^	50.00 ± 1.58^**^
MCV (FL)	61.40 ± 1.67	61.20 ± 1.10	59.20 ± 1.92	59.80 ± 1.92
MCH (PG)	21.00 ± 0.00	21.20 ± 0.45	19.00 ± 0.71^**^	19.60 ± 0.55^**^
MCHC (G/DL)	34.20 ± 1.10	34.20 ± 0.45	31.80 ± 0.45^**^	32.60 ± 0.55^**^
Platelet count (X 10^9^/L)	798.40 ± 83.94	763.80 ± 114.88	956.80 ± 212.12	1001.80 ± 25.68^**^
WBC (X 10^9^/L)	5.46 ± 1.96	7.44 ± 0.68	7.82 ± 3.26	9.82 ± 1.70^**^
Neutrophil (%)	18.40 ± 7.89	9.80 ± 2.86	11.00 ± 3.46	15.20 ± 8.64
Lymphocyte (%)	75.60 ± 6.58	85.80 ± 3.96^**^	84.00 ± 3.81	79.60 ± 7.09
Monocyte (%)	6.00 ± 2.12	4.40 ± 1.14	4.60 ± 2.70	5.20 ± 4.21
Eosinophil (%)	0.00 ± 0.00	0.00 ± 0.00	0.40 ± 0.89	0.00 ± 0.00
Basophil (%)	0.00 ± 0.00	0.00 ± 0.00	0.00 ± 0.00	0.00 ± 0.00
Atypical lymphocyte (%)	0.00 ± 0.00	0.00 ± 0.00	0.00 ± 0.00	0.00 ± 0.00

*Values are expressed in mean ± SD (n = 5/group)*.

** *Significant difference with control (ANOVA, Dunnett's t (two-sided) test, p < 0.05)*.

**Table 3:** **Serum biochemical values of male and female rats treated with 250, 500, and 1000 mg/kg of *L. tigris* E sclerotial powder for 28-days**.

**Parameter**	**Treatment**			
	**Control**	**1000 mg/kg**	**500 mg/kg**	**250 mg/kg**
**MALE**				
Glucose (mmol/L)	6.00 ± 0.77	6.00 ± 1.08	6.24 ± 0.68	6.66 ± 1.63
Urea (mmol/L)	6.44 ± 0.93	6.58 ± 0.98	5.38 ± 1.17	4.94 ± 0.21^**^
Creatinine (μmol/L)	15.20 ± 2.86	15.40 ± 3.65	12.00 ± 3.39	16.60 ± 4.04
Calcium (mmol/L)	2.52 ± 0.10	2.50 ± 0.05	2.37 ± 0.09^**^	2.49 ± 0.08
Inorganic phosphate (mmol/L)	3.75 ± 0.21	3.26 ± 0.35	3.11 ± 0.36^**^	3.17 ± 0.50
Uric acid (mmol/L)	0.11 ± 0.07	0.09 ± 0.02	0.07 ± 0.05	0.08 ± 0.06
Sodium (mmol/L)	145.80 ± 1.48	145.60 ± 1.14	146.20 ± 1.92	146.00 ± 2.00
Potassium (mmol/L)	4.48 ± 0.36	4.28 ± 0.38	4.84 ± 1.04	5.24 ± 1.00
Chloride (mmol/L)	107.00 ± 1.87	106.00 ± 0.71	109.20 ± 2.59	109.80 ± 1.48
Total cholesterol (mmol/L)	2.32 ± 0.47	2.10 ± 0.33	1.40 ± 0.16^**^	1.00 ± 0.48^**^
Total protein (G/L)	57.80 ± 2.59	58.20 ± 3.70	44.68 ± 22.08	54.60 ± 3.05
Albumin (G/L)	36.60 ± 1.95	37.20 ± 1.92	34.40 ± 1.52	34.60 ± 1.82
Globulin (G/L)	21.20 ± 0.84	21.00 ± 2.00	20.00 ± 1.00	20.00 ± 1.41
Total bilirubin (μmol/L)	1.80 ± 0.45	2.00 ± 0.00	2.00 ± 0.00	2.00 ± 0.00
Alkaline phosphatase (IU/L)	196.40 ± 42.44	169.20 ± 31.14	215.80 ± 20.85	177.00 ± 20.20
SGOT (AST) (IU/L)	239.00 ± 46.48	254.60 ± 24.63	252.00 ± 32.83	244.00 ± 43.00
SGPT (ALT) (IU/L)	61.20 ± 7.50	59.20 ± 5.22	65.60 ± 8.26	67.40 ± 8.44
GGT (IU/L)	1.80 ± 1.30	0.00 ± 0.00	0.00 ± 0.00	0.00 ± 0.00
**FEMALE**				
Glucose (mmol/L)	6.08 ± 0.37	6.48 ± 0.99	6.60 ± 0.51	7.28 ± 0.70
Urea (mmol/L)	7.48 ± 1.28	8.66 ± 1.24	7.08 ± 0.26	7.86 ± 0.72
Creatinine (μmol/L)	16.60 ± 5.13	18.00 ± 1.87	23.80 ± 4.15	23.60 ± 6.35
Calcium (mmol/L)	2.43 ± 0.05	2.36 ± 0.06	2.52 ± 0.07	2.66 ± 0.11^**^
Inorganic phosphate (mmol/L)	2.83 ± 0.28	2.75 ± 0.27	3.40 ± 0.39	3.96 ± 0.50^**^
Uric acid (mmol/L)	0.06 ± 0.01	0.06 ± 0.01	0.07 ± 0.01	0.07 ± 0.01
Sodium (mmol/L)	142.40 ± 0.89	143.60 ± 1.14	144.80 ± 1.10	144.00 ± 2.55
Potassium (mmol/L)	4.04 ± 0.70	3.84 ± 0.30	4.90 ± 0.37^**^	5.98 ± 0.59^**^
Chloride (mmol/L)	104.00 ± 1.41	106.60 ± 1.82	105.80 ± 0.45	105.40 ± 1.67
Total cholesterol (mmol/L)	2.16 ± 0.29	2.02 ± 0.27	1.62 ± 0.47	1.90 ± 0.37
Total protein (G/L)	56.00 ± 2.92	55.00 ± 1.87	58.60 ± 2.61	61.40 ± 4.83
Albumin (G/L)	35.60 ± 1.82	34.80 ± 1.48	37.40 ± 1.34	39.80 ± 3.70
Globulin (G/L)	20.40 ± 1.14	20.20 ± 0.84	21.20 ± 1.79	21.60 ± 1.34
Total bilirubin (μmol/L)	1.60 ± 0.55	2.00 ± 0.00	1.00 ± 0.00	1.00 ± 0.00
Alkaline phosphatase (IU/L)	131.20 ± 20.17	155.00 ± 40.74	144.80 ± 28.49	115.40 ± 22.78
SGOT (AST) (IU/L)	212.20 ± 19.20	188.60 ± 21.95	242.20 ± 23.88	295.00 ± 28.06^**^
SGPT (ALT) (IU/L)	55.80 ± 6.30	54.20 ± 8.79	56.40 ± 3.97	61.00 ± 2.83
GGT (IU/L)	2.00 ± 0.71	0.40 ± 0.55	1.40 ± 2.61	1.60 ± 1.14

*Values are expressed in mean ± SD (n = 5/group)*.

** *Significant difference with control (ANOVA, Dunnett's t (two-sided) test, p < 0.05)*.

Serum biochemistry analysis showed statistically significant differences in a few parameters in both treated males and females. Urea level in males of the 250 mg/kg group was significantly lower than the control group. The males of the 500 mg/kg group showed lower level (*p* < 0.05) of calcium and inorganic phosphate compared to control group. Similarly, total cholesterol level was also significantly lower in the 250 and 500 mg/kg male rats. In the treated female rats, the 250 mg/kg group exhibited higher level (*p* < 0.05) of calcium, inorganic phosphate, potassium, and SGOT compared to the control group. High potassium level was also observed in the females of the 500 mg/kg group (*p* < 0.05). These alterations attained statistical significance in the mid-dose and lowest-dose groups only and did not follow a dose-dependent pattern.

### Relative organ weights

Relative organ weights of the treated and control rats are shown in Table 4. The relative organ weight of each organ of both male and female rats in the treatment groups did not show any significant differences (*p* > 0.05) compared to the control.

**Table 4:** **Relative organ weight of male and female rats treated with *L. tigris* E sclerotial powder for 28-days**.

**Organ**	**Treatment**							
	**Male**				**Female**			
	**Control**	**1000 mg/kg**	**500 mg/kg**	**250 mg/kg**	**Control**	**1000 mg/kg**	**500 mg/kg**	**250 mg/kg**
Heart	0.380 ± 0.027	0.369 ± 0.032	0.386 ± 0.019	0.356 ± 0.010	0.332 ± 0.170	0.371 ± 0.015	0.410 ± 0.049	0.372 ± 0.041
Lungs	0.390 ± 0.019	0.352 ± 0.030	0.355 ± 0.018	0.383 ± 0.076	0.440 ± 0.030	0.433 ± 0.030	0.594 ± 0.153	0.472 ± 0.025
Liver	3.062 ± 0.235	2.818 ± 0.328	2.750 ± 0.123	2.793 ± 0.206	3.272 ± 0.190	3.12 ± 0.259	2.975 ± 0.249	3.238 ± 0.180
Spleen	0.208 ± 0.010	0.196 ± 0.017	0.189 ± 0.011	0.208 ± 0.017	0.196 ± 0.021	0.21 ± 0.044	0.238 ± 0.029	0.224 ± 0.027
Right kidney	0.349 ± 0.032	0.331 ± 0.040	0.345 ± 0.028	0.356 ± 0.019	0.363 ± 0.038	0.341 ± 0.025	0.350 ± 0.021	0.388 ± 0.055
Left kidney	0.340 ± 0.026	0.324 ± 0.042	0.332 ± 0.017	0.353 ± 0.009	0.359 ± 0.033	0.340 ± 0.017	0.338 ± 0.026	0.376 ± 0.048
Prostate + seminal vesicle + glands/ uterus	0.659 ± 0.045	0.609 ± 0.038	0.688 ± 0.027	0.710 ± 0.079	0.231 ± 0.050	0.239 ± 0.094	0.221 ± 0.049	0.162 ± 0.018
Right testis/ovary	0.436 ± 0.035	0.481 ± 0.053	0.482 ± 0.040	0.430 ± 0.037	0.025 ± 0.003	0.023 ± 0.003	0.030 ± 0.004	0.023 ± 0.003
Left testis/ovary	0.434 ± 0.023	0.476 ± 0.038	0.471 ± 0.031	0.418 ± 0.027	0.030 ± 0.006	0.026 ± 0.003	0.032 ± 0.005	0.025 ± 0.004
Right epididymis	0.154 ± 0.010	0.160 ± 0.011	0.161 ± 0.009	0.166 ± 0.009	−	−	−	−
Left epididymis	0.148 ± 0.011	0.159 ± 0.010	0.16 ± 0.006	0.162 ± 0.011	−	−	−	−

Values are expressed in mean ± SD (n = 5/group). There was no significance difference in the relative organ weight between the control and treated rats (p > 0.05).

### Histopathology analysis

**Figure 2:**
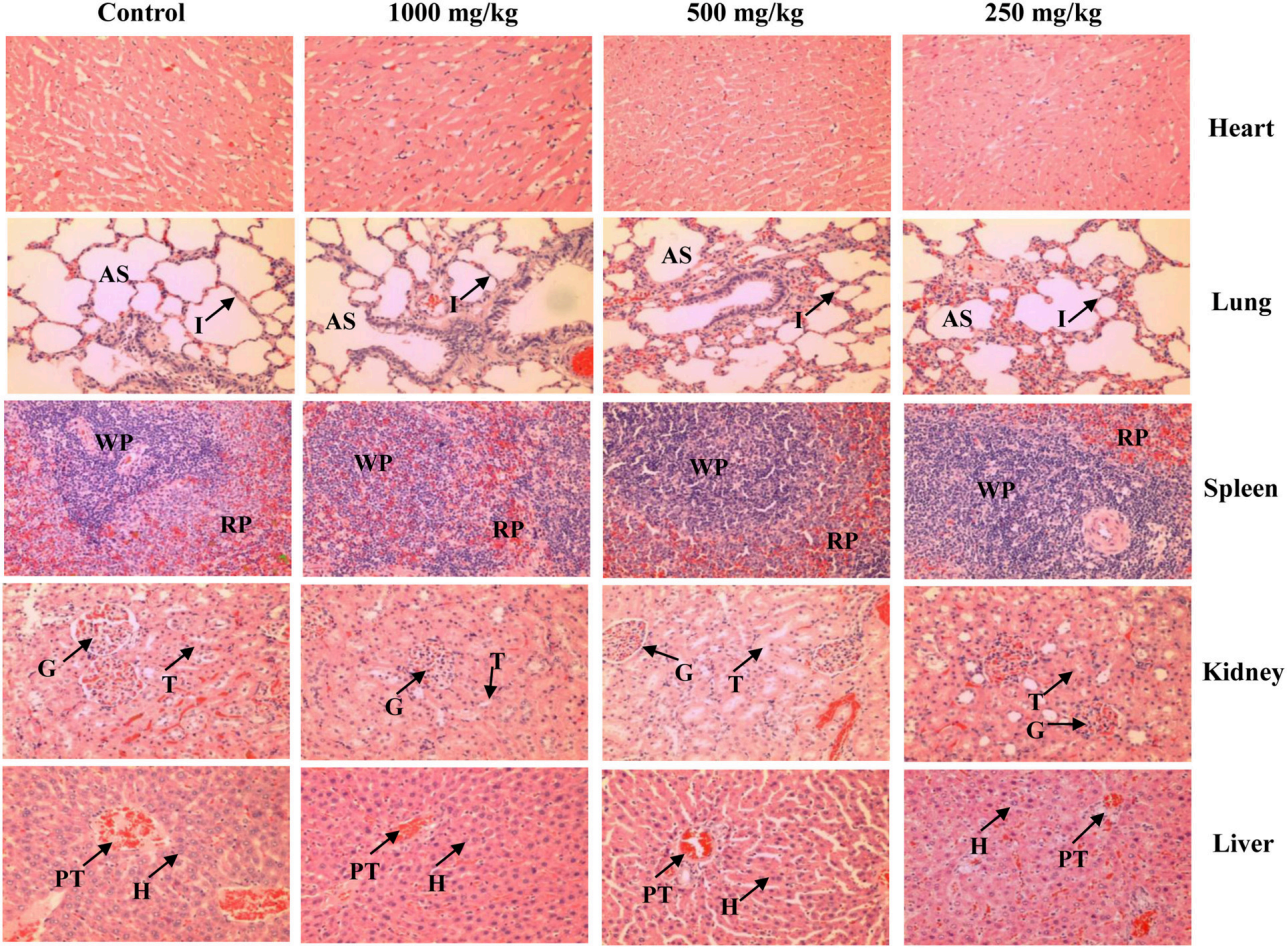
**Representative photos of hematoxylin and eosin stain (100 × magnification) of vital organs (heart, lung, spleen, kidney, and liver) of the control and treated rats (250, 500, and 1000 mg/kg of *L. tigris* E sclerotial powder)**. No significant histopathological abnormality was observed in the organs of the control and treated rats. AS, alveolar space; I, interstitium; WP, white pulp; RP, red pulp; G, glomerulus; T, tubule; PT, portal tract; H, hepatocyte.

**Figure 3:**
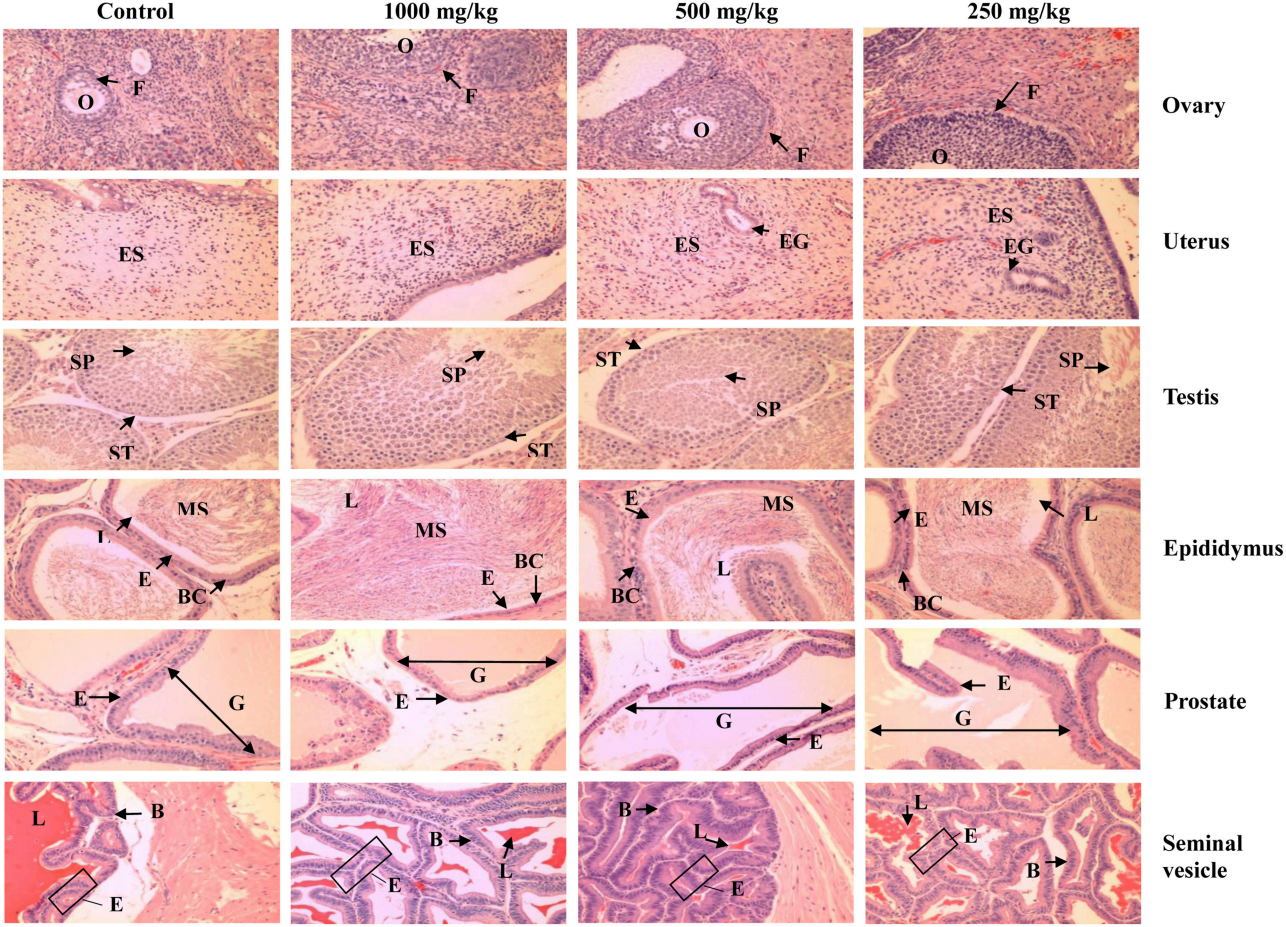
**Representative photos of hematoxylin and eosin stain (100 × magnification) of reproductive organs (ovary, uterus, testis, epididymis, prostate, and seminal vesicle) of the control and treated rats (250, 500, and 1000 mg/kg of *L. tigris* E sclerotial powder)**. No significant histopathological abnormality was observed in the organs of the control and treated rats. O, oocyte; F, follicle; ES, endometrial stroma; EG, endometrial gland; ST, seminiferous tubule; SP, spermatozoids; MS, mature sperm; E, epithelium; L, lumen; BC, basal cells; G, gland.

Light microscopic examination of the vital organs including heart, lung, liver, spleen, kidney, ovary, uterus, testis, epididymis, prostate, and seminal vesicle of the rats in all the treated and control groups did not reveal any gross pathological lesions. No significant differences were found in the histological examination of the organ sections in the tissue of rats treated with 250, 500, and 1000 mg/kg of *L. tigris* E sclerotial powder as compared to those of controls. The histological sections of heart, lung, liver, spleen, kidney, ovary, uterus, testis, epididymis, prostate, and seminal vesicle of the control and treated rats are illustrated in Figures 2, 3. In both the control and treated female and male rats, the heart shows normal cardiac muscle fibers and lungs show normal alveolar structure with no treatment-related inflammatory response. No histopathological changes were observed in the kidneys and liver. Normal glomeruli, tubules and interstitial space were observed in the kidneys, while normal architecture, hepatocytes, and portal tract with no congestion of sinusoids and fatty changes were seen in the liver. Similarly, normal structure and histology of the spleen were also observed in all the rats. Although mild congestion was observed in the lung, liver, and kidney of the treated and control groups of both sexes, these findings were incidental or spontaneous with no relation to *L. tigris* E sclerotial powder treatment. Administration of *L. tigris* E sclerotial powder at a daily dose of up to 1000 mg/kg for 28 consecutive days also did not showed any toxic effects on the reproductive systems of the rats. Histological analysis on the rat testes showed normal seminiferous tubules, spermatogonia, spermatocytes, and interstitial tissues; epididymis shows normal epithelial and basal cells; prostate shows normal glands and interstitial; and seminal vesicle shows normal glands. The reproductive organs of the female rats (ovary and uterus) showed normal histological structures.

## Discussion

The present study showed the safety profile of *L. tigris* E sclerotium in sub-acute oral toxicity study done on SD rats. The results of this 28-day toxicity study revealed no significant adverse effects following oral administration of *L. tigris* E sclerotial powder at 3 different doses (250, 500, and 1000 mg/kg). Changes in body weight and internal organs have been used as a reference to indicate the harmful effects from exposure to potentially toxic substances of drugs and chemicals (Teo et al., 2002; Hilaly et al., 2004; Mukinda and Eagles, 2010). In this study, all the rats at the respective dosage groups showed a similar growth pattern (Figure 1), and continued normal weight gain (Table 1) throughout the 28-day toxicological study. In addition, there is no significant differences in the relative organ weights (Table 4) between the male and female rats in all the treated and control groups. This indicated that there is no gross toxic effect due to oral administration of *L. tigris* E sclerotial powder at a daily dose up to 1000 mg/kg for 28 consecutive days.

The hematopoietic system is a sensitive target for toxic substances and changes in hematological parameters provide high predictive value of toxicity or pathological status in human using the data obtained from animal model study (Olson et al., 2000; Adeneye et al., 2006). In this study, alterations in the hematological parameters of the rats treated with the *L. tigris* E sclerotial powder did not produce any treatment-associated hematopoiesis toxicity. Although there are significant decrease of MCV in 250 and 500 mg/kg groups, decrease of MCH in 250 mg/kg group and decrease of neutrophil percentage in 500 mg/kg group of the treated male rats, but the differences were not dose-related or reflected by any changes in other related parameters. With respect to the observed changes in hematology parameters in the treated female rats, including the increase of RBC, decrease of MCH and MCHC in 250 and 500 mg/kg groups; increase of hemoglobin and PCV in 250 and 1000 mg/kg groups; increase of platelet and WBC in 250 mg/kg group, and increase of lymphocyte in 1000 mg/kg group, the differences were assumed to be toxicologically irrelevant because the values were within the normal range of laboratory reference data (Petterino and Argentino-Storino, 2006; Han et al., 2010). Hence, the variations observed in the hematological parameters were considered to be unrelated with the administration of *L. tigris* E sclerotial powder because the changes were neither the sex nor the dose-related and the correlative findings in the organ weight and histopathological analyses were absent.

In addition to the hematological analysis, clinical biochemistry data of the blood also plays an important role in determination of the toxicity induced by drugs and chemical substances. Biochemical parameters for total protein, albumin, globulin, total bilirubin, alkaline phosphatase, SGOT, SGPT, and GGT are good indicators of hepatic functions; while urea, creatinine, and uric acid levels are good indicators of renal functions. In the present study, significant differences (*p* < 0.05) in the SGOT were observed in females of the 250 mg/kg treatment group. The changes were not dose-related and are unlikely to be of any clinical significance as the other hepatic function test parameters did not show any significant differences. Moreover, there were no lesions or pathological changes were observed in the liver of the rats, indicating that *L. tigris* E sclerotial powder did not cause hepatotoxicity or deterioration of the liver function.

Elevations in serum urea, creatinine, and electrolytes (potassium, inorganic phosphate, sodium, and chloride) levels are relatively sensitive indicators for renal injury. This study showed that there are no significant changes in the level of urea and creatinine in all the treated rats with the exception of the urea level in males of low dose 250 mg/kg treatment group which is slightly lower (*p* < 0.05) than the rats of the control group, which is likely an incidental event. Although, an increase of serum potassium concentrations (*p* < 0.05) was observed in the middle dose (500 mg/kg) and low dose (250 mg/kg) of the female rats, the values were within the normal range (Petterino and Argentino-Storino, 2006; Han et al., 2010). Differences in inorganic phosphate (*p* < 0.05) (decrease in males of the 500 mg/kg treatment group and increase in females of the 250 mg/kg treatment group) were neither the sex nor the dose-related. Other serum electrolyte parameters in all the treated rats are not significantly different from the control rats. As the differences of the serum urea, inorganic phosphate and potassium are unlikely to be of any clinical significance and no pathological changes was found in the glomeruli, tubules and interstitial space structures of kidney tissue in treated rats, therefore the administration of *L. tigris* E sclerotial powder up to 1000 mg/kg for 28 consecutive days has no deleterious effects on the renal function.

The gross examinations of the vital organs of rats treated with different dosage forms of *L. tigris* E sclerotial powder did not show any differences in color and texture compared with the organs of the control rats. Besides, no hypertrophy of organs were observed in this study, indicating no toxic effects of *L. tigris* E sclerotial powder on the rats. Microscopic examinations revealed no changes in the cell structures or pathologies in the heart, lungs, liver, kidneys, spleen, and the reproductive organs including uterus, ovaries, testes, prostate, and seminal vesicle of the treated rats. The histological examinations are in good supportive correlation with the findings from hematological and clinical biochemistry studies. It showed that *L. tigris* E sclerotial powder did not cause any remarkable alteration or damages on the blood cells, serum and the vital organs.

The sclerotium of the Tiger Milk mushrooms, *Lignosus* spp. have been consumed by the indigenous people and local communities as traditional medicine in the Southeast Asia and China for its medicinal properties and or as general tonic. *L. tigris* was recently discovered as distinct from its other more well-known species, the *L. rhinocerotis* and has subsequently been successfully cultivated. Our earlier study (Kong et al., 2016) showed that the mushroom sclerotia are nutritive and contain numerous bioactive compounds, such as phenolics, terpenoids, and glucans which are known to contain anti-cancer, anti-inflammatory, antioxidant, and immunomodulatory activities. In summary, this 28-day sub-acute toxicity study has successfully demonstrated the safety profile of the *L. tigris* E sclerotia. The results obtained in the study suggest that *L. tigris* E sclerotial powder is not toxic at all the tested dosages (250, 500, and 1000 mg/kg), and did not produce any signs of toxicity or showed any evidence of systems disruptions as tested. The NOAEL dose for sub-acute toxicity of the *L. tigris* E sclerotial powder is more than 1000 mg/kg. This data indicates that the mushroom is safe to consume at the dose up to 1000 mg/kg. However, further evaluations on the chronic toxicity, effect on fertility, teratogenicity and mutagenicity are necessary to further validate the safety consumption of this mushroom.

## Author contributions

BK performed the experiments, analyzed the data, and drafted the manuscript. JP helped to perform and analyzed histopathological data. NT and SF conceived and designed the study. NT and SF helped to draft the manuscript.

## Funding

This research is supported by FRGS grant FP029-2014A from University of Malaya. BK is supported by Postgraduate Research Grant (PPP) PG144-2014B from University of Malaya, Malaysia.

### Conflict of interest statement

The authors declare that the research was conducted in the absence of any commercial or financial relationships that could be construed as a potential conflict of interest.
